# The natural course of otitis media with effusion in infants who failed universal newborn hearing screening: a retrospective cohort study

**DOI:** 10.1017/S0022215123000452

**Published:** 2023-10

**Authors:** Y-L Hu, Z-F Xia, W-B Tuo, C-H Yuan, W-N Guo, C Yao

**Affiliations:** 1 Department of Otolaryngology, Wuhan Children's Hospital, Tongji Medical College, Huazhong University of Science & Technology, Wuhan, PR China; 2 Department of Laboratory Medicine, Wuhan Children’s Hospital, Tongji Medical College, Huazhong University of Science & Technology, Wuhan, PR China; 3 Health Care, Wuhan Children's Hospital, Tongji Medical College, Huazhong University of Science and Technology, Wuhan, PR China

**Keywords:** Infant, otitis media with effusion, hearing loss

## Abstract

**Objectives:**

To analyse the natural course of infants with otitis media with effusion who failed universal newborn hearing screening and to explore the appropriate observation period.

**Methods:**

This retrospective cohort analysis included infants with otitis media with effusion who failed universal newborn hearing screening every 3 months for 12 months.

**Results:**

The average recovery time of the 155 infants was 7.08 ± 0.32 months after diagnosis. Multivariate Cox regression analysis confirmed that frequent reflux, maxillofacial deformities and initial hearing status were independent factors affecting recovery. Moreover, the cumulative recovery of most infants with mild hearing loss and infants with moderate hearing loss accompanied by frequent reflux was significantly higher at six months after diagnosis than at three months.

**Conclusion:**

For most infants with mild hearing loss, as well as those with moderate hearing loss accompanied by frequent reflux, the observation period can be extended to six months after diagnosis.

## Introduction

Universal newborn hearing screening protocols have been widely applied worldwide in recent decades. The Joint Committee on Infant Hearing has established benchmarks recommending that all neonates be screened for hearing loss before the age of one month.^1^ Those not passing the screening should complete a diagnostic evaluation prior to three months of age, and those identified with permanent hearing loss should begin audiological, medical and educational interventions before they are six months of age. In China, the timing of hearing screening, diagnosis and intervention is consistent with this standard.^2^

In neonates who failed universal newborn hearing screening, 15–65 per cent of cases were caused by otitis media with effusion (OME).^3^ According to the American Clinical Practice Guideline: Otitis Media with Effusion (Update),^4^ two recommendations apply to these infants. For infants aged six months or older with documented bilateral OME for at least three months and documented hearing difficulties, clinicians should offer a tympanostomy tube. For those who decline this procedure, follow-up appointments should be continued until the effusion is no longer present or until surgical intervention. At present, the surgical treatment timing for these infants mostly adheres to this guideline, with an operation rate of about 50 per cent.^5^ One study even suggested that a diagnostic microscopic examination with myringotomy, with or without placement of a ventilation tube, should be performed.^6^

As a leading institution for the treatment of children with ENT diseases in Wuhan, China, we provide clinical services for a large number of children with OME. Our observations indicate that few parents are willing to approve surgery after a three-month observation period because of concerns over the adverse effects of general anaesthesia and complications of myringotomy with or without ventilation tube placement.^7^ Therefore, they tend to prolong the observation time. A small pilot study conducted by our institute found that infants with OME had a high rate of self-healing, and long-term observation did not affect post-operative outcomes.^8^

Currently, however, there is limited research on the natural course of infants with OME. This study retrospectively analysed the natural course of infants with OME who failed universal newborn hearing screening and who were observed at follow up, in order to provide guidance for determining the most suitable period for observation and follow up.

## Materials and methods

### Study design and patient selection

**Figure 1.**
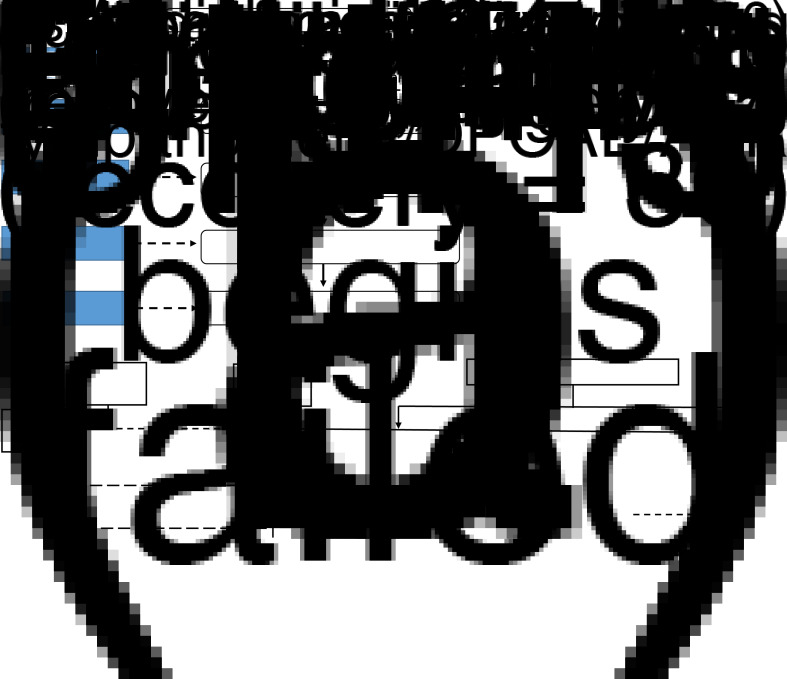
Research profile and design for clinical and audiology assessment in children with otitis media with effusion (OME). (a) Study flow chart of enrolment and follow up of children with OME. (b) Time course of audiology assessment and outcome evaluation. UNHS = universal newborn hearing screening; DPOAE = distortion-product otoacoustic emissions; ABR = auditory brainstem responses

This retrospective cohort study enrolled infants referred to our hospital's otolaryngology hearing centre because of failed universal newborn hearing screening during the period from 2009 to 2019. Initially, 170 children were included in the study; 15 children were excluded because of incomplete data and other reasons, and 155 children were finally included in the study. The research proposal and process are displayed in Fig. 1.

Infants who failed universal newborn hearing screening and who were previously diagnosed with otitis media with effusion (OME) before three months of age were included in the study. Follow up was conducted every 3 months, for a duration of 12 months, until the effusion had resolved or surgery was performed. Auditory function tests were conducted at each follow-up appointment, and any accompanying symptoms were documented.

Based on the main causes of secretory otitis media^4^ and the physicians’ clinical diagnoses, the associated symptoms were grouped into major symptom clusters, such as recurrent respiratory infections (viral or bacterial),^9^ maxillofacial deformities (e.g. cleft palate, Down syndrome), frequent nasal congestion (nasal congestion without recurrent respiratory infections, or mouth breathing) and frequent reflux^10^ (vomiting, reflux or extra-oesophageal symptoms). Patients with no obvious accompanying symptoms were classified as ‘uncertain’. Furthermore, symptomatic treatment with medication was not restricted.

The exclusion criteria were as follows: (1) sensorineural hearing loss; (2) deformity of the pinna, external auditory canal or middle ear; (3) history of ear trauma; and (4) history of ear canal effusion.

### Diagnostic criteria

The diagnostic criteria for OME were as follows: (1) presence of middle-ear effusion on pneumatic otoscopy; (2) abnormal results of higher-frequency probe tone (1 kHz) tympanometry; (3) failed distortion-product otoacoustic emission responses; and (4) abnormal air conduction auditory brainstem response (ABR) thresholds and normal bone conduction ABR thresholds.^4^

### Hearing classification criteria

There is no clear classification of hearing loss in infants. In this study, the hearing levels were classified by the air conduction ABR threshold of the worse hearing ear: class I (25–30 dB nHL), class II (35–40 dB nHL), class III (45–50 dB nHL) and class IV (55–60 dB nHL).^11^ Classes I and II are mild, and classes III and IV are moderate. Normal clinical and audiological examination findings for both ears was designated ‘recovery’.

### Statistical methods

Continuous variables with normal distribution, such as age at diagnosis and average recovery time, are presented as mean ± standard deviation. Patients were allocated to different subgroups based on discrete variables (e.g. sex, initial hearing level, accompanying symptoms), which are presented as frequency and constituent ratio (f, per cent). The cumulative recovery rates at different follow-up timepoints, average recovery time and median recovery time were documented for the overall group and each subgroup. Survival curve analysis was used to describe differences in the cumulative recovery rates among the overall group and each subgroup at different follow-up timepoints.

The Kaplan–Meier method and Breslow test were used for comparison of recovery status between subgroups. Univariate and multivariate Cox regression analyses were applied to analyse recovery according to the hazard ratio for different initial features. Because respiratory infections can affect the resolution time of middle-ear effusion,^4^ infants with recurrent respiratory infections were used as controls in regression analysis of influencing factors. The chi-square test was used to analyse the changes in recovery rate between different follow-up timepoints for patients with different initial characteristics and their combinations.

All statistical analyses were performed using R software (version 4.2.0; R Foundation for Statistical Computing, Vienna, Austria) and GraphPad Prism (version 8.0; GraphPad Software, San Diego, California, USA). A two-tailed *p*-value of less than 0.05 was the criterion for statistical significance.

## Results

### Basic characteristics of infants

Among the 155 infants with otitis media with effusion (OME), 102 were male (65.8 per cent) and 53 were female (34.2 per cent), and the average age at initial diagnosis was 2.83 ± 0.28 months. As shown in Table 1, the main accompanying symptoms in descending order were frequent reflux (26.4 per cent), frequent nasal congestion (25.2 per cent), recurrent respiratory infections (16.8 per cent) and maxillofacial deformities (11.6 per cent), while there were 31 children (20.0 per cent) with no obvious associated symptoms. Initial hearing testing indicated that infants with OME had different degrees of hearing loss (class I, 17.4 per cent; class II, 37.4 per cent; class III, 35.5 per cent; class IV, 9.7 per cent).

**Table 1.** Basic characteristics of infants with OME

Characteristic	Cases (*n* (%))
Sex	
– Male	102 (65.8)
– Female	53 (34.2)
Accompanied symptoms	
– Frequent reflux	41 (26.4)
– Frequent nasal congestion	39 (25.2)
– Recurrent respiratory infections	26 (16.8)
– Maxillofacial deformities	18 (11.6)
– Uncertain	31 (20.0)
Initial hearing level	
– Class I (25–30 dBnHL)	27 (17.4)
– Class II (35–40 dBnHL)	58 (37.4)
– Class III (45–50 dBnHL)	55 (35.5)
– Class IV (55–60 dBnHL)	15 (9.7)

OME = otitis media with effusion

### Average recovery time

The mean recovery time of the total infant group was 7.08 ± 0.32 months in this study, and the median recovery time was 6 (interquartile range, 4.80 - 7.20) months. The cumulative recovery rate reached 39.4 per cent, 58.7 per cent, 65.8 per cent and 69.7 per cent at the 3-month, 6-month, 9-month and 12-month follow-up timepoints, respectively. Thirty-five cases (22.6 per cent) were not yet healed after follow up, and in 12 cases (7.7 per cent) surgery was opted for.

The analysis of mean recovery time for different characteristics showed no significant difference between male and female patients (7.41 ± 0.40 months *vs* 6.45 ± 0.52 months, *p* = 0.31), while the results for infants with different main accompanying symptom clusters varied significantly (*p* < 0.01). Mean recovery times of infants with frequent reflux (4.61 ± 0.38 months) and no obvious accompanying symptoms (‘uncertain’, 5.32 ± 0.64 months) were lower than those of infants with frequent nasal congestion, recurrent respiratory infections or maxillofacial deformities. Infants with maxillofacial deformities had the longest mean time to recovery (11.83 ± 0.16 months).

**Figure 2.**
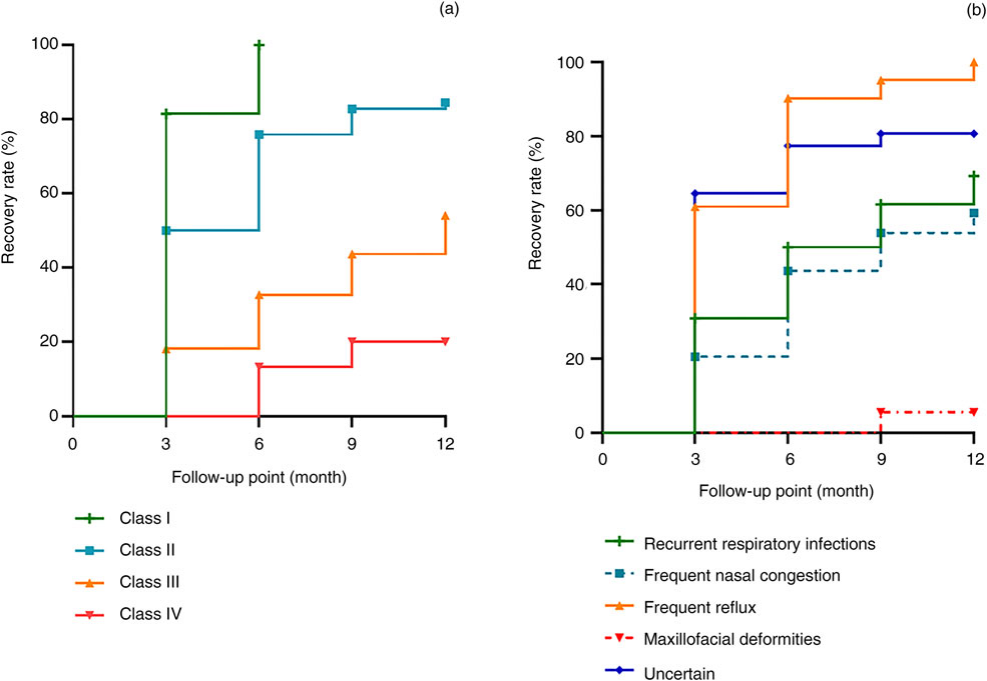
The cumulative self-healing rate of infants at various follow-up timepoints, according to different characteristics. (a) The cumulative self-healing rate of otitis media with effusion (OME) infants with different initial hearing levels. (b) The cumulative self-healing rate of OME infants with different initial symptoms.

Mean recovery time for infants with different initial hearing levels showed an upward trend with the aggravation of hearing loss (*p* < 0.01), as shown in Table 2. The cumulative recovery rates of children with different levels of hearing loss at each timepoint were significantly different at the three-month follow up, and this difference persisted until the end of the follow up. The cumulative recovery rates, in order of frequency, were highest for class I, class II, class III and class IV hearing loss (Fig. 2a).

**Table 2.** Comparison of mean time to recovery in OME infants

Characteristic	Time to recovery (mean (95% CI); months)	χ^2^	*P*
Sex		1.04	0.31
– Male	7.41 (6.64–8.19)		
– Female	6.45 (5.44–7.46)		
Initial symptoms		54.72	<0.01
– Recurrent respiratory infections	7.73 (6.22–9.25)		
– Frequent nasal congestion	8.46 (7.29–9.63)		
– Frequent reflux	4.61 (3.87–5.35)		
– Maxillofacial deformities	11.83 (11.52–12.15)		
– Uncertain	5.32 (4.07–6.57)		
Initial hearing level (dBnHL)		64.06	<0.01
– Class I	3.56 (3.11–4.00)		
– Class II	5.74 (4.87–6.06)		
– Class III	9.16 (8.20–10.13)		
– Class IV	11 (9.94–12.06)		

OME = otitis media with effusion; CI = confidence interval

In addition, the recovery rates of the infants with frequent reflux (61.1 per cent) and ‘uncertain’ symptoms (64.5 per cent) were higher than those of the other three groups at the three-month follow up, and the recovery rate of those with frequent reflux increased significantly at the six-month follow up and exceeded that of asymptomatic patients. It is noteworthy that the recovery rate among subgroups was ranked in descending order as frequent reflux, ‘uncertain’ symptoms, recurrent respiratory infections, frequent nasal congestion and maxillofacial deformities, and this order of ranking persisted from this point until the end of follow up. Infants with maxillofacial deformities had the lowest recovery rate, and there was no change except in one case, which was resolved at nine months and throughout the subsequent follow-up period (Fig. 2b).

### Regression analysis

This section concerns the regression analysis of the influence of risk factors on hearing recovery. Based on the results displayed in Table 2 and Fig. 2, accompanying symptoms and hearing levels are strongly associated with recovery rates and duration of recovery. The Cox regression model analysing time to recovery status (Yes = 1) was applied to further explore the risk of initial symptoms and hearing level on hearing recovery.

As shown in Table 3, univariate Cox regression results indicated that reflux, maxillofacial deformities and initial hearing status are the factors affecting recovery. Compared with recurrent respiratory infections, frequent reflux may accelerate recovery, while maxillofacial deformities have the opposite effect, with a hazard ratio of 2.18 (95 per cent confidence interval (CI), 1.24–3.84) and 0.06 (95 per cent CI, 0.01–0.44), respectively. Meanwhile, level II or poorer initial hearing loss may delay the recovery of children compared with level I hearing loss (*p* < 0.05). Results from the multivariate Cox regression model stratified by age and sex further confirmed frequent reflux, maxillofacial deformities and initial hearing status as independent factors affecting recovery.

**Table 3.** Hazard ratios for hearing recovery by different symptoms on univariate and multivariate regression

Character	Univariate Cox regression		Multivariate Cox regression*	
	HR (95% CI)	*P*-value	HR (95% CI)	*P*-value
Initial symptom				
– Contrast (recurrent respiratory infections = yes)				
– Frequent nasal congestion	0.79 (0.43–1.47)	0.46	0.88 (0.47–1.64)	0.69
– Frequent reflux	2.18 (1.24–3.84)	0.01	1.97 (1.10–3.50)	0.02
– Maxillofacial deformities	0.06 (0.01–0.44)	0.01	0.07 (0.01–0.56)	0.01
– Uncertain	1.64 (0.89–3.01)	0.11	1.29 (0.67–2.45)	0.44
Initial hearing level				
– Contrast (class I)				
– Class II	0.56 (0.34–0.90)	0.02	0.49 (0.29–0.82)	0.01
– Class III	0.24 (0.14–0.42)	<0.01	0.33 (0.18–0.60)	<0.01
– Class IV	0.08 (0.02–0.26)	<0.01	0.13 (0.04–0.49)	<0.01

* Multiple cox regression adjust by gender and age. HR = hazard ratio; CI = confidence interval

### Variations in cumulative recovery rates

This section concerns the variation in cumulative recovery rates for infants with OME at different timepoints. Independent risk factors affecting hearing recovery (Table 3) were reclassified according to their attributes. Initial symptoms were re-categorised into frequent reflux, maxillofacial deformities and others (including frequent nasal congestion, recurrent respiratory infections and ‘uncertain’ (i.e. no obvious accompanying symptoms)), while the initial degree of hearing loss remained unadjusted. The recovery rates at each timepoint were compared according to different combinations of initial symptoms and degrees of hearing loss.

Comparison of recovery rate changes revealed that, for infants with reflux and class I, II or III hearing loss, the cumulative recovery at the six-month follow up was significantly higher than at the three-month follow up (*p* < 0.05). However, the change between the six- and nine-month follow-up assessments was not statistically significant (*p* > 0.05), as the infants with level II initial hearing loss had all recovered at the nine-month follow up. For infants with other initial symptoms and class I or II initial hearing loss, the cumulative recovery at the six-month follow up was significantly higher than at the three-month follow up (*p* < 0.05); the recovery rate change between the six- and nine-month follow-up appointments was not statistically significant. Moreover, comparisons of the cumulative recovery rates at the 3-month versus 6-month, 6-month versus 9-month, and the 9-month versus 12-month follow-up timepoints all showed no significant differences for infants with level III initial hearing loss. None of the infants with OME accompanied by maxillofacial deformities healed throughout follow up, except for one case that was resolved at the nine-month follow up. For children with class IV hearing loss, the conclusion was not statistically significant because of insufficient sample size in this study (Table 4).

**Table 4.** Cumulative recovery rates of OME infants at follow up, by initial symptoms and hearing levels

Initial symptom	Initial hearing level	Cases (*n*)	Cumulative recovery (f (%))*			
			3 months	6 months	9 months	12 months
Frequent reflux	Class I	5	4 (80.0)	5 (100.0)		
	Class II	28	19 (67.9)	26 (92.9)	28 (100.0)	
	Class III	7	2 (28.6)	5 (71.4)	5 (71.4)	7 (100.0)
	Class IV	1	0 (0.0)	1 (100.0)		
Maxillofacial deformities	Class II	1	0 (0.0)	0 (0.0)	0 (0.0)	0 (0.0)
	Class III	12	0 (0.0)	0 (0.0)	1 (8.3)	1 (8.3)
	Class IV	5	0 (0.0)	0 (0.0)	0 (0.0)	0 (0.0)
Others	Class I	22	18 (81.8)	22 (100)	22 (100)	22 (100)
	Class II	29	10 (34.5)	18 (62.1)	20 (69.0)	21 (72.4)
	Class III	36	8 (22.2)	13 (36.1)	18 (50)	21 (58.3)
	Class IV	9	0 (0.0)	1 (11.1)	2 (22.2)	2 (22.2)

* Frequency and constituent ratio. OME = otitis media with effusion

## Discussion

For infants diagnosed with otitis media with effusion (OME) and failed universal newborn hearing screening, our study found that the total cumulative recovery rate increased to varying degrees with the extension of follow-up time, and the range of hearing loss was mainly mild to moderate. Considering that the age of 12–18 months is a critical period for speech development,^4^ the impact of an appropriate extension of follow-up time will be acceptable if it is less likely to have a serious impact on the child's speech. Thus, there are feasible and worthwhile benefits to extending the follow-up period to six months or even longer for most infants with OME. However, other results of this study showed differences in recovery rate and recovery time among groups with different accompanying symptoms and initial hearing levels. Therefore, decisions regarding the duration of follow-up observation must be based on the individual's clinical characteristics.

It is necessary to first analyse the aetiology of OME. In research on the source of neonatal middle-ear effusion, previous evidence has shown amniotic fluid cellular content in the middle-ear cavity of newborns; although most of it practically resolved around 5 months after birth, tiny remnants of amniotic fluid cellular content appeared as late as 15 months after birth.^12^ Therefore, such infants may appear to have failed the universal newborn hearing screening and then receive a diagnosis of OME if effusion remains after audiological diagnosis within three months after birth. According to the resolution time of the amniotic fluid cellular content in the middle-ear cavity and the self-healing time of children with OME, the clinical practice guidelines suggest that observation for three months after diagnosis is reasonable. Our study reported a recovery rate of 39.4 per cent at three months of observation, indicating that middle-ear effusions of most of the infants were not completely resolved.

In our study, infants with different concomitant symptoms showed different recovery times and recovery rates. Respiratory tract infection is the main risk factor for acute otitis media, and OME may occur during or after an upper respiratory tract infection.^4^ Resolution of middle-ear effusion after acute infection can be prolonged, with 50 per cent of cases resolved within one month and about 80 per cent after three months.^13^

Studies have confirmed that fluid can pass through the Eustachian tube into the middle ear during swallowing.^14^ Reflux will develop in 70–80 per cent of infants within two months of age; most cases will resolve after seven months of age, and 95 per cent will be eliminated by the age of one year.^10^ In our study, the hearing recovery rate was greater than 60 per cent and greater than 90 per cent at the follow-up periods of three months and six months, respectively. This was consistent with the natural course that Rosenfeld and Kay reported for untreated otitis media, in which children with reflux reached a 60 per cent symptom resolution rate within three months and a 75 per cent resolution rate within six months.^13^

In addition to respiratory tract infection, long-term nasal congestion in infants is mostly related to allergies. Antihistamines can be used after six months of age, according to the clinical practice guidelines for allergic rhinitis developed by the American Academy of Otolaryngology–Head and Neck Surgery Foundation.^15^ In this study, the improvement in recovery rate for children with OME after six months of follow up may be related to the improvement of nasal congestion in some such infants. However, the self-healing rate of infants with long-term nasal obstruction is lower than that of infants with recurrent respiratory infections and reflux, and this may be due to frequent recurrence of rhinitis, poor symptom control and other factors such as adenoid hypertrophy.^16^

Compared with recurrent respiratory infections, maxillofacial deformities serve as a risk factor for recovery. Infants with maxillofacial deformities are prone to OME, which is not only closely related to gastroesophageal reflux but also to Eustachian tube dysfunction and poor resistance.^4^ It has been reported that more than 20 per cent of middle-ear effusions resolved after the repair of maxillofacial deformities.^17^ However, other studies suggest early or exceptionally early tympanostomy tube placement.^18^

Regular audiological evaluation is also valuable.^4^ The ABR thresholds of the infants in this study ranged from 25 to 60 dB nHL, which is consistent with the study findings of Boudewyns *et al*.^19^ Studies have shown that the average hearing loss in OME is 18–35 dB HL,^20^ and the degree of hearing loss is highly positively correlated with effusion volume. This study's results revealed that infants with mild hearing loss had higher recovery rates and shorter recovery times. Initial hearing status was one of the independent factors affecting recovery. Therefore, assessment of initial hearing levels is another important indicator used to guide decisions to continue observation.

In this study, the recovery rates at each timepoint were compared according to different combinations of initial symptoms and degrees of hearing loss. Except for those with maxillofacial deformities, the cumulative recovery of all infants with mild hearing loss and those with reflux accompanied by moderate hearing loss was significantly higher at six months after diagnosis than at three months. Therefore, the observation period can be extended up to six months after diagnosis for these infants; however, ventilation tubes can be placed in a timely way for infants with maxillofacial deformities and most with moderate hearing levels. Because the age of 12–18 months is a critical period for speech development,^4^ the maximum period of observation in children with hearing loss should not exceed this.

- In infants who fail universal newborn hearing screening, 15–65 per cent of cases are due to otitis media with effusion (OME)
- At present, the surgical treatment rate of infants aged six months or older is approximately 50 per cent
- In this study, most infants with OME who failed universal newborn hearing screening recovered within 12 months of diagnosis
- Observation duration in follow up can be determined based on degree of hearing loss and accompanying symptoms
- For most infants with mild hearing loss, or moderate hearing loss accompanied by frequent reflux, the observation period can be extended to six months after diagnosis
- Infants with maxillofacial deformities and most infants with moderate hearing loss were less likely to self-heal

Therefore, clinicians must comprehensively analyse the relevant causes of OME, predict the possibility of early recovery, and guide the feeding, nursing and disease observation methods for these infants.^4^ As a retrospective analysis, this study mainly analysed the possible aetiologies through the main accompanying symptoms documented in the case records, which may be limited. In addition, infants with recurrent respiratory infections were used as controls in regression analysis of influencing factors. Further controlled studies will provide more evidence for the analysis of risk factors in infants with OME.
